# No More Need Apply

**Published:** 1894-03

**Authors:** 


### NO MORE NEED APPLY.

The General Medical Council of Great Britain has settled the matter for those who commenced their course of study at Harvard and Michigan Universities previously to the passage of the law rejecting all American diplomas.

This is unquestionably the only consistent course, although it bears severely on the young men who had honestly endeavored to fulfil the requirements of the law. It is very evident that the editor of the journal of the British Dental Association voices the sentiment of English dentists when he plaintively says, "To us the applications prove how urgent was the necessity for a change in the administration of this portion of the Dentists' Act." He evidently anticipated a mob of young American dentists, and was ready to give thanks that the deluge had been averted.

